# Efficacy and Safety of Vacuum-Assisted Excision for Benign Breast Mass Lesion: A Meta-Analysis

**DOI:** 10.3390/medicina57111260

**Published:** 2021-11-17

**Authors:** Hee-Seon Yoo, Wu-Seong Kang, Jung-Soo Pyo, Junghan Yoon

**Affiliations:** 1 Department of Surgery, Mizpia Hospital, Gwangju-si 61963, Gyeonggi-do, Korea; zinbang38@naver.com; 2 Department of Trauma Surgery, Cheju Halla General Hospital, Jeju-si 63127, Jeju-do, Korea; wuseongkang@naver.com; 3 Department of Pathology, Uijeongbu Eulji Medical Center, Eulji University School of Medicine, Dongil-ro, Uijeongbu-si 11759, Gyeonggi-do, Korea; 4 Department of Surgery, Chonnam National University Hwasun Hospital, Hwasun-eup 58128, Jeollanam-do, Korea

**Keywords:** breast mass, vacuum assisted excision, meta-analysis, systematic review

## Abstract

*Background and Objective*: Breast mass lesions are common; however, determining the malignant potential of the lesion can be ambiguous. Recently, to evaluate breast mass lesions, vacuum-assisted excision (VAE) biopsy has been widely used for both diagnostic and therapeutic purposes. This study aimed to investigate the therapeutic role of VAE. *Materials and Methods*: Relevant articles were obtained by searching PubMed and EMBASE on 3 September 2021. Meta-analyses were performed using odds ratios and proportions. To assess heterogeneity, we conducted a subgroup analysis and meta-regression tests. *Results*: Finally, 26 studies comprising 18,170 patients were included. All of these were observational studies. The meta-analysis showed that the complete resection rate of VAE was 0.930. In the meta-regression test, there was no significant difference. The meta-analysis showed a recurrence rate of 0.039 in the VAE group. The meta-regression test showed no statistical significance. Postoperative hematoma, pain, and ecchymosis after VAE were 0.092, 0.082, and 0.075, respectively. *Conclusion*: VAE for benign breast lesions showed favorable outcomes with respect to complete resection and complications. This meta-analysis suggested that VAE for low-risk benign breast lesions is a reasonable option for both diagnostic and therapeutic purposes.

## 1. Introduction

Breast mass lesions are very common, but it can be difficult to determine their malignant potential [1]. Because breast cancer is one of the leading causes of mortality worldwide, it is crucial to detect malignant lesions [1,2]. Although the performance of the imaging system to evaluate the potential of malignancy has increased, biopsy is the final informative method for diagnosis [3]. Histologic confirmation is enabled by fine-needle aspiration, core needle biopsy, open excision, and vacuum-assisted excision (VAE) biopsy. Among these modalities, fine-needle aspiration and core needle biopsy can only be used for diagnostic purposes, but it has some limitations [3]. Fine-needle aspiration and core needle biopsy cannot examine the whole mass, whereas VAE can perform a full examination of the entire mass. Owing to the advantage that VAE can remove the entire mass region, VAE has recently been widely used for both diagnostic and therapeutic purposes [3,4,5]. In 2002, the US Food and Drug Administration (FDA) approved VAE for the removal of benign lesions. Although VAE can remove a large amount of tissue, open surgical excision remains the gold standard for large palpable masses [6]. Thus, the indications and efficacy of VAE remain controversial. Although traditional open excision is the most effective method for removing all mass lesions, it results in scarring, which is not preferred by women. Most surgeons would respect this patient’s preference if the procedure is secure.

This study aimed to investigate the efficacy and safety of VAE. We investigated the complete resection rate, recurrence rate, and complications after VAE for benign breast mass lesions.

## 2. Materials and Methods

### 2.1. Published Study Search and Selection Criteria

This study was performed in accordance with the Preferred Reporting Items for Systematic Reviews and Meta-Analyses [7]. Relevant articles were obtained by searching PubMed and EMBASE on 3 September 2021. These databases were searched using the following keywords: “(breast) AND (benign) AND (vacuum-assisted OR mammotome) AND (excision)”. The titles and abstracts of all searched articles were screened for exclusion. Review articles and meta-analyses were screened to obtain additional eligible studies. Search results were then reviewed, and articles were included if the study investigated vacuum-assisted biopsy for benign breast tumor.

The inclusion criteria for the present review were as follows: (1) patients with a benign breast tumor, (2) patients who underwent vacuum-assisted biopsy, (3) study comprised relevant outcomes such as operative and postoperative measurements, and (4) odds ratio (OR) or proportional data reported, or data provided for their calculation. Articles that studied other diseases, non-original articles, or non-English language publications were excluded. Complete resection was defined as no remnant lesion identified on postoperative or postprocedural sonographic examination. Recurrence was defined as a newly occurring lesion within the previously excised region.

### 2.2. Data Extraction

Data from all eligible studies were extracted by two investigators. Extracted data from each of the eligible studies included the following: the first author’s name, year of publication, study location, study design, study period, number of patients analyzed, age, number of lesions, indication of vacuum-assisted biopsy, complete resection rate, recurrence rate, and complications.

### 2.3. Quality Assessment

We used the Newcastle-Ottawa quality assessment scale (NOS) to assess the risk of bias in observational studies [8]. NOS uses a star system that includes three domains: selection, comparability, and exposure/outcome. All studies were independently reviewed by two investigators. Any disagreement concerning study selection and data extraction was resolved by consensus.

### 2.4. Statistical Analysis

To obtain the estimated effect sizes, a meta-analysis was performed using the Comprehensive Meta-Analysis software package (Biostat, Englewood, NJ, USA). We computed the point estimate by combining single descriptive statistics to pool the overall proportions [9]. Pooled proportion of event was estimated using fixed-effect and random-effect model of meta-analysis. To pool the proportion (complete resection, recurrence, and complications), we used logit-transformed values to avoid the squeezing of variance effect [10,11]. Confidence interval was calculated using the exact confidence limits for a binominal proportion [11]. To pool the odds ratio for binary data, we used the inverse variance method with random effects weighing for meta-analysis of outcomes. As the eligible studies used populations with heterogeneity, a random-effects model was more appropriate than a fixed-effects model. Heterogeneity between eligible studies was checked using Cochran’s Q (Chi-square test) (*p*-value < 0.10 were considered significant). Egger’s test was conducted to evaluate the publication bias.

We performed a subgroup analysis to assess the heterogeneity across the studies. The pooled incidence of complete resection, recurrence, and complications was calculated according to study-level characteristics as follows: (1) mass removal technique (VAE or surgical open excision) (2) breast tumor size. We also conducted a meta-regression test for each moderator to assess heterogeneity.

## 3. Results

### 3.1. Selection and Characteristics

**Figure 1:**
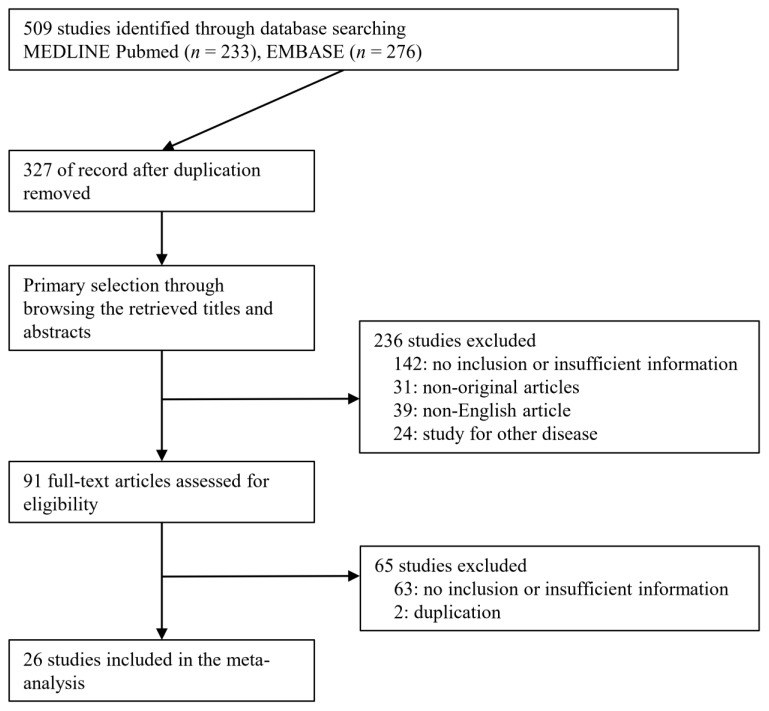
Flowchart summarizes literature and study selection.

A total of 509 studies were identified through a database search. Among the searched studies, 236 were excluded. Studies were excluded because they were non-original (*n* = 31), studied other diseases (*n* = 24), or were written in a non-English language (*n* = 39). Finally, 26 studies [12,13,14,15,16,17,18,19,20,21,22,23,24,25,26,27,28,29,30,31,32,33,34,35,36,37] comprising 18,170 patients were included in the present meta-analysis (Figure 1), and detailed information about the eligible studies is shown in Table 1.

All were observational studies, and there were no randomized controlled trials (Table 1). Two studies included benign phyllodes tumors [31,32]. The indications of 16 studies vary from BI-RADS 1-4b [11,16,17,18,19,20,22,23,24,28,30,31,32,33,34,37]. Perez-Fuentes et al. include eight patients with BI-RADS 5 [13] Hahn et al. included two patients with BI-RADS 5 [23] Jiang et al. included 48 patients with BI-RADS 5 in cases where patients desired biopsy [30]. Sixteen studies were conducted in Asian countries [14,18,19,20,22,24,25,27,28,29,30,31,32,34,36,37].

### 3.2. Quality Assessment

The quality assessment and risk of bias for each eligible study are summarized in Table 2. All the included studies were observational. The NOS score of 11 studies (42.3%) was 4 points (lowest) and that of nine studies (34.6%) was 7 points (highest). We found that all studies had insufficient selection of controls in the selection domain and non-response rate in the exposure domain. Overall, substantial confounding factors may exist with respect to selection and exposure. Particularly, only four studies directly compared VAE with open excision.

### 3.3. Complete Resection and Recurrence Rate

**Figure 2:**
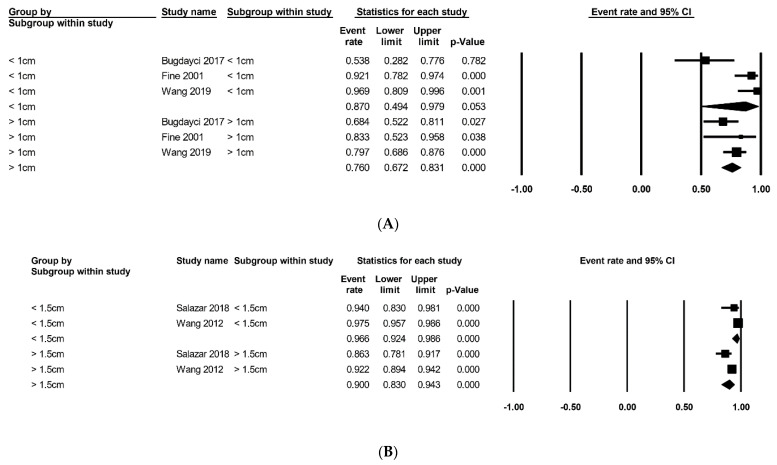
The estimated rates of complete resection in vacuum-assisted excision based on tumor size. (**A**) According to 1 cm of tumor size; (**B**) According to 1.5 cm of tumor size.

The estimated rates of complete resection and recurrence through vacuum-assisted and open excisions are summarized in Table 3. The meta-analysis showed that the complete resection rate of VAE was 0.930 (95% confidence interval [CI], 0.897–0.954; Cochran’s Q, *p* < 0.001; Egger’s test, *p* = 0.566) [13,15,16,17,18,20,22,23,24,25,26,27,28,33,34,35,36,37]. In the meta-regression test, there was no significant difference (*p* = 0.154). The meta-analysis showed a recurrence rate of 0.039 (95% CI, 0.016–0.091; Cochran’s Q, *p* < 0.001; Egger’s test, *p* = 0.243) in the VAE group [21,29,30,31,32]. The meta-regression test showed no statistical significance (*p* = 0.896). Subgroup analysis according to tumor size is summarized in Figure 2. Three studies comprised subgroups that were divided by 1.0 cm of tumor size (Figure 2A) [12,33,37]. The estimated rates of complete resection of the subgroup with a tumor size <1.0 cm was 0.870 (95% CI, 0.494–0.979; Cochran’s Q, *p* = 0.002; Egger’s test, *p* = 0.515), whereas that of the subgroup with a tumor size > 1.0 cm was 0.760 (95% CI, 0.672–0.831; Cochran’s Q, *p* = 0.359). The meta-regression test showed no statistical significance (*p* = 0.489). Two studies comprised subgroups that were divided by 1.5 cm tumor size (Figure 2B) [28,35]. The estimated rates of complete resection of the subgroup with a tumor size <1.5 cm was 0.966 (95% CI, 0.924–0.986; Cochran’s Q, *p* = 0.163), whereas that of the subgroup with a tumor size > 1.0 cm was 0.900 (95% CI, 0.830–0.943; Cochran’s Q, *p* = 0.060). The meta-regression test showed statistical significance (*p* = 0.021).

**Figure 3:**
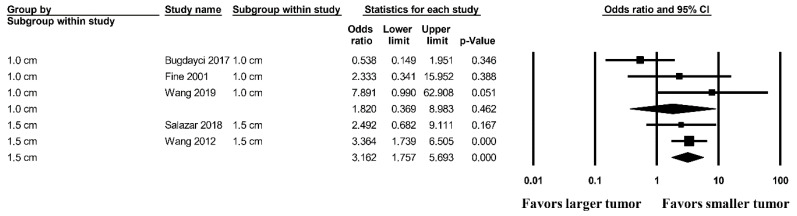
The odds ratio for complete resection in vacuum-assisted excision based on tumor size group.

Figure 3 shows the OR for complete resection according to tumor size [12,28,33]. Smaller tumors (<1.5 cm) showed a more favorable complete resection rate than those larger than 1.5 cm (OR, 3.162; 95% CI, 1.757–5.693; *p* < 0.001). However, in the criterion of 1.0 cm of tumor size, there was no significant difference (OR, 1.829; 95% CI, 0.369–8.983; *p* = 0.462) between larger and smaller tumors.

### 3.4. Pooled Incidence of Postoperative Hematoma, Pain, and Ecchymosis

Postoperative hematomas, pain, and ecchymosis are summarized in Table 4. The estimated incidence of postoperative hematoma in VAE was 0.092 (95% CI, 0.067–0.126; Cochran’s Q, *p* < 0.001; Egger’s test 0.668), whereas that after open excision was 0.015 (95% CI, 0.003–0.073; Cochran’s Q, *p* = 0.305; meta-regression test, *p* = 0.049) [14,15,16,17,18,19,20,21,22,23,24,25,28,29,30,33,35,37]. The estimated incidence of postoperative pain was 0.082 (95% CI, 0.049–0.134; Cochran’s Q, *p* < 0.001; Egger’s test, *p* = 0.004) [15,16,18,19,21,23,24,25,26,28,29,33,35,37]. The estimated incidence of postoperative ecchymosis was 0.075 (95% CI, 0.045–0.115; Cochran’s Q, *p* < 0.001; Egger’s test, *p* = 0.046) [15,16,18,19,20,21,23,24,25,26,28,29,37].

## 4. Discussion

Our analysis suggested that benign breast masses could be safely removed using VAE. The estimated complete resection rate is sufficiently high. Indeed, the estimated rates of recurrence and complications after VAE were sufficiently low. The complete resection and recurrence rate are critical to the use of both diagnostic and therapeutic procedures. However, a limited number of comparative studies and high heterogeneity indicate that more prospective comparative studies are warranted. Nonetheless, our study indicates that VAE is a useful option for successfully removing benign breast masses. However, in terms of high-risk lesions such as phyllodes tumor or atypical ductal hyperplasia, there have been insufficient studies to demonstrate oncologic safety.

VAE was approved by the FDA in 2002 for the removal of benign lesions and by the National Institute for Health and Care Excellence in the United Kingdom in 2006 [5]. However, it is still controversial for some high-risk lesions, such as phyllodes tumors or atypical lesions [5]. To date, several guidelines recommend VAE as an alternative to conventional open excision for benign breast lesions. The international consensus reference in Swiss [6] recommends minimally invasive management of selected B3 lesions with therapeutic VAE. However, according to this consensus, open surgery is still the gold standard for atypical ductal hyperplasia and phyllodes tumors. The study group of breast ultrasonography in Germany [38] stated that VAE allows the resection of breast tissue up to 8 cm^3^ (volume). However, it has not been formulated as a nationwide guideline. In the statement of the American Society of Breast Surgeons, it was recommended that easily visualized, confirmed histologically prior to treatment, and less than 4 cm are indications of VAE for fibroadenoma [39]. All these guidelines allow the use of VAE for breast masses that are limited to low-risk lesions.

During our literature search, we found four comparative studies, in which VAE was compared to open excision [14,20,31,32]. All of these studies were observational. Two studies [31,32] comprised benign phyllodes tumors, whereas two [14,20] comprised other benign breast masses such as fibroadenoma, fibrocystic change, papilloma, atypical ductal hyperplasia, or fibrocystic nodule. However, Chen et al. [14] did not report the complete resection rate of all benign masses, in which the complete excision rate of breast carcinomas was reported. Wang et al. [20] reported a 3.4% incomplete removal rate in the VAE group, which was not significantly different from that of open excision. Two studies [31,32] regarding benign phyllodes tumors reported no significant difference in recurrence rate (2.4% [in VAE] vs. 0% [in open excision] [32]), (11% [in VAE] vs. 6.8% [in open excision] [31]). Complete excision and recurrence rates of VAE were favorable. Although VAE for phyllodes tumors is still controversial [38], our review suggests that the curability of VAE as an alternative to surgery is promising.

Recently, a meta-analysis of 15 studies involving 5256 patients reported the efficacy and safety of VAE [40]. This study showed no significant difference in tumor size, postoperative hematomas, ecchymosis, or residual disease between VAE and open excision. However, the authors used the Chinese database, and many studies are written in the Chinese language. All eligible studies were conducted in China. Only two studies could be searched using the Medline database. This may induce substantial language or location bias [41]. We excluded Chinese-written literature from our analysis. Our study is an updated meta-analysis comprising more recent studies. Another systematic review was conducted without a meta-analysis that aimed to investigate VAE for fibroadenoma [42]. However, this study has some crucial limitations, including the small number of studies (*n* = 4), no assessment of risk of bias, and no pooling estimates.

Our analysis has several limitations. First, all eligible studies were not randomized trials but were observational. There was no prospective study. Selection bias was inevitable. Second, we used single descriptive statistics because there were limited comparative studies. This may have resulted in substantial heterogeneity. To overcome this issue, we conducted subgroup analysis and meta-regression. Third, among the eligible studies, the indication of VAE was substantially heterogeneous, and high-risk or moderate-risk masses were not excluded. Fourth, we included limited a number of studies with open excision because there was a limited number of comparative studies between VAE and open excision. Therefore, the certainty of the comparison of effect size was limited in our meta-analysis. Instead, we noted the single proportional effect size of VAE. Fifth, in Egger’s test, publication bias existed regarding two outcomes of pain and ecchymosis. However, other crucial outcomes such as complete resection or recurrence had no publication bias in Egger’s Test. In our study, we did not include unpublished studies such as grey literature, dissertation, or conference presentation. In general, studies with significance are more likely to be published [9]. This may contribute to publication bias. Nevertheless, studies that have not been in peer-reviewed journals tend to be of lower quality [9]. We acknowledge that our study may have potential publication bias regarding pain and ecchymosis, but we believe these complications have a less clinical impact than other outcomes. Finally, only articles written in English were included. Overall, our study has a substantial risk of bias due to the nature of a non-randomized study and non-comparative study. The publication bias implies that there might exist more complications. The limitation of our studies emphasizes the need for future study that is comparative with open excision prospectively. Furthermore, it is needed to compare other minimally invasive procedures to remove breast mass. Recently, some procedures such as cryoablation, microwave ablation, high-intensity focused ultrasonography, or laser therapy were introduced and some have shown promising results [5]. However, they have still limited evidence and only cryoablation and VAE have received FDA approval [5]. In the future study, systematic review for these procedures would be needed.

## 5. Conclusions

In conclusion, VAE for benign breast lesions showed favorable outcomes with respect to complete resection and complications. This meta-analysis suggested that VAE for low-risk benign breast lesions is a reasonable option for both diagnostic and therapeutic purposes. However, a limited number of comparative studies on open excision have weakened the strength of evidence. More comparative prospective studies are required to estimate the true effect size.

## Figures and Tables

**Table 1:** Main characteristics of the eligible studies.

	Study, Year	Location	Study Design	Study Period	Number of Patients	Number of Lesions	Tx	Dx/Lesion	Indication	Subgroup	Gauge
1	Fine 2001 [12]	USA, single center	Observational	1999–1999	45	50	VAE	Variable	Visualized breast lesion on mammography or ultrasonography		11
2	Perez-Fuentes 2001 [13]	Venezuela, single center	Observational	1998–2000	83	88	VAE	Variable	BI-RADS Category 2–5		11
3	Chen 2003 [14]	Taiwan, single center	Observational	1998–2001	128		VAE	Variable	Non palpable lesion		11
					104		OE	Variable			
4	Fine 2003 [15]	USA, multicenter	Observational	ND	216		VAE	Variable	Low risk palpable mass	8	8
										11	11
5	Alonso-Bartolome 2004 [16]	Spain, single center	Observational	2000–2002	97	102	VAE	Variable	BI-RADS Category 3		11
6	Krainick-Strobel 2007 [17]	Germany, single center	Observational	2000–2003	45	46	VAE	Variable	BI-RADS Category 3		8 or 11
7	Ko 2008 [18]	Korea, single center	Observational	2002–2003	199	263	VAE	Variable	BI-RADS Category 3		8 or 11
8	Wang 2009 [19]	China, single center	Observational	2007–2008	244		VAE	Variable	BI-RADS Category 3	Benign	10
9	Wang 2009 [20]	China, single center	Observational	2004–2006	62	150	VAE	Variable	BI-RADS Category 1–3		8 or 11
					36	87	OE	Variable			
10	Maxwell 2009 [21]	UK, single center	Observational	2002–2008	1		VAE	Papilloma	papilloma	EnCor	7
					12			Papilloma	papilloma	Mammotome	8
					13			Papilloma	papilloma	Mammotome	11
11	Yom 2009 [22]	Korea, single center	Observational	2001–2004	150		VAE		BI-RADS Category 2–4	Within 2 yr	8
					184		VAE			After 2 yr	8
12	Hahn 2009 [23]	Germany, single center	Observational	2006–2007				Variable	BI-RADS Category 3–5	Overall	
					21		VAE	Variable		Mammotome	8
					17		VAE	Variable		ATEC	9
					9		VAE	Variable		Mammotome	11
					15		VAE	Variable		ATEC	12
13	Wang 2010 [24]	China, single center	Observational	April 2008–September 2008	79		VAE	Variable	BI-RADS Caterfory 3 or benign by previous core needle biopsy		
					35		VAE	Variable			7
					44		VAE	Variable			10
14	Chang 2011 [25]	Korea, single center	Observational	2007–2009	83		VAE	Papillary tumor	Papillary tumor	Overall	11
							VAE			Benign	11
							VAE			Atypical	11
							VAE			Malignant	11
15	Slanetz 2011 [26]	USA, single center	Observational	2003–2005	40	42	VAE	Variable	fibroadenoma, probably benign nodule		Variable
16	Youk 2011 [27]	Korea, single center	Observational	2007–2009	62		VAE	Variable	Benign papilloma without atypia	Benign	8 or 11
17	Wang 2012 [28]	China, single center	Observational	2005–2009					BI-RADS Category 3, 4a, 4b	Overall	
					484		VAE	Variable		Vacora	10
					143		VAE	Variable		Mammotome	8
					356		VAE	Variable		EnCor	7
										<1.5 cm	
										> = 1.5 cm	
18	Li 2013 [29]	China, single center	Observational	2007–2009	1578	3854		Variable	benign lesion		8
19	Jiang 2013 [30]	China, single center	Observational	2008–2012	3681	4867	VAE	Variable	BI-RADS Category 1–4, 5 if patient desired		8
20	Ouyang 2015 [31]	China, single center	Observational	2005–2013	108		VAE	Benign Phllyodes tumor	BI-RADS Category 3, 4		ND
					117		OE	Benign Phllyodes tumor			
21	Kim 2016 [32]	Korea, single center	Observational	2002–2012	126		OE	Benign Phllyodes tumor	BI-RADS Category 3, 4		
					20		VAE	Benign Phllyodes tumor			11 or 8
22	Bugdayci 2017 [33]	Turkey, single center	Observational	1999–2001	51			Fibroadenoma	BI-RADS Category 3, 4A	Overall	11
					13					<1 cm	
					32					1–2 cm	
					6					2–3 cm	
23	Park 2018 [34]	Korea, single center	Observational	2003–2015	8748	11,221	VAE	Variable	BI-RADS Category 3, 4A		8
24	Salazar 2018 [35]	Spain, single center	Observational	2012–2016	143	152	VAE		benign lesion	After tx	10 or 7
										FU 6 mo	
										<16 mm	
										> = 16 mm	
25	Choi 2019 [36]	Korea, single center	Observational	2005–2015	233		VAE		Benign papilloma without atypia		11 or 8
					206		OE				
26	Wang 2019 [37]	China, single center	Observational	2008–2016	101		VAE	Intraductal papilloma	BI-RADS Category 3, 4A, 4B		7
										< 1 cm	
										> = 1 cm	

Abbreviation; BI-RADS, breast imaging, reporting and data system; VAE, vacuum assisted excisional biopsy; OE, open excision; ND, non-descriptive.

**Table 2:** NOS for the risk of bias and quality assessment of NRSs.

Study No.	Author, Year	Selection				Comparability	Exposure			Total Score
		Adequate Definition of Patient Cases	Representativeness of Patient Cases	Selection of Controls	Definition of Controls	Control for Important or Additional Factors	Ascertainment of Exposure	Same Method of Ascertainment for Participants	Nonresponse Rate	
1	Fine 2001 [12]	⋆	⋆	⋆	⋆	⋆	⋆	⋆		7
2	Perez-Fuentes 2001 [13]	⋆	⋆	⋆			⋆			4
3	Chen 2003 [14]	⋆	⋆	⋆	⋆	⋆	⋆	⋆		7
4	Fine 2003 [15]	⋆	⋆	⋆		⋆	⋆			5
5	Alonso-Bartolome 2004 [16]	⋆	⋆				⋆	⋆		4
6	Krainick-Strobel 2007 [17]	⋆	⋆				⋆	⋆		4
7	Ko 2008 [18]	⋆	⋆	⋆			⋆	⋆		5
8	Wang 2009 [19]	⋆	⋆				⋆	⋆		4
9	Wang 2009 [20]	⋆	⋆	⋆	⋆	⋆	⋆	⋆		7
10	Maxwell 2009 [21]	⋆	⋆	⋆			⋆	⋆		5
11	Yom 2009 [22]	⋆	⋆				⋆	⋆		4
12	Hahn 2009 [23]	⋆	⋆	⋆			⋆	⋆		5
13	Wang 2010 [24]	⋆	⋆	⋆	⋆	⋆	⋆	⋆		7
14	Chang 2011 [25]	⋆	⋆	⋆	⋆	⋆	⋆	⋆		7
15	Slanetz 2011 [26]	⋆	⋆				⋆	⋆		4
16	Youk 2011 [27]	⋆	⋆				⋆	⋆		4
17	Wang 2012 [28]	⋆	⋆				⋆	⋆		4
18	Li 2013 [29]	⋆	⋆	⋆	⋆	⋆	⋆	⋆		7
19	Jiang 2013 [30]	⋆	⋆				⋆	⋆		4
20	Ouyang 2015 [31]	⋆	⋆	⋆	⋆	⋆	⋆	⋆		7
21	Kim 2016 [32]	⋆	⋆	⋆	⋆	⋆	⋆	⋆		7
22	Bugdayci 2017 [33]	⋆	⋆	⋆	⋆	⋆	⋆	⋆		7
23	Park 2018 [34]	⋆	⋆	⋆	⋆		⋆	⋆		6
24	Salazar 2018 [35]	⋆	⋆				⋆	⋆		4
25	Choi 2019 [36]	⋆	⋆	⋆	⋆	⋆	⋆	⋆		7
26	Wang 2019 [37]	⋆	⋆				⋆	⋆		4

NOS, Newcastle–Ottawa scale; NRS, non-randomized study; ⋆, The study has met the criteria for a domain of the Newcastle–Ottawa Scale.

**Table 3:** The estimated rates of complete resection and recurrence through vacuum-assisted and open excisions.

Subgroup	Number of Subsets	Fixed Effect [95% CI]	Heterogeneity Test [*p*-Value]	Random Effect [95% CI]	Egger’s Test [*p*-Value]	Meta-Regression Test [*p*-Value]
Complete resection, rate						
Vacuum-assisted excision	23	0.935 [0.931, 0.940]	<0.001	0.930 [0.897, 0.954]	0.566	0.154
Open excision	1	0.994 [0.907, 1.000]	1.000	0.994 [0.907, 1.000]	-	
Recurrence, rate						
Vacuum-assisted excision	5	0.018 [0.015, 0.021]	<0.001	0.039 [0.016, 0.091]	0.243	0.896
Open excision	2	0.051 [0.028, 0.090]	0.110	0.044 [0.016, 0.119]	-	

CI, Confidence interval.

**Table 4:** The estimated complication rates after vacuum-assisted and open excision.

Subgroup	Number of Subsets	Fixed Effect [95% CI]	Heterogeneity Test [*p*-Value]	Random Effect [95% CI]	Egger’s Test [*p*-Value]	Meta-Regression Test [*p*-Value]
Hematoma						
Vacuum-assisted excision	25	0.088 [0.082, 0.095]	<0.001	0.092 [0.067, 0.126]	0.668	0.049
Open excision	2	0.015 [0.003, 0.073]	0.305	0.015 [0.003, 0.075]	-	
Pain						
Vacuum-assisted excision	21	0.224 [0.210, 0.240]	<0.001	0.082 [0.049, 0.134]	0.004	
Open excision	0					
Ecchymosis						
Vacuum-assisted excision	21	0.142 [0.130, 0.156]	<0.001	0.075 [0.045, 0.115]	0.046	0.297
Open excision	1	0.014 [0.001, 0.182]	1.000	0.014 [0.001, 0.182]	-	

CI, Confidence interval.

## Data Availability

Data is contained within the article.
